# The Detection of Invisible Abnormal Metabolism in the FDG-PET Images of Patients With Anti-LGI1 Encephalitis by Machine Learning

**DOI:** 10.3389/fneur.2022.812439

**Published:** 2022-05-30

**Authors:** Jian Pan, Ruijuan Lv, Guifei Zhou, Run Si, Qun Wang, Xiaobin Zhao, Jiangang Liu, Lin Ai

**Affiliations:** ^1^School of Computer and Information Technology, Beijing Jiaotong University, Beijing, China; ^2^Department of Neurology, Beijing Tiantan Hospital, Capital Medical University; China National Clinical Research Center for Neurological Diseases, Beijing, China; ^3^Department of Nuclear Medicine, Beijing Tiantan Hospital, Capital Medical University, Beijing, China; ^4^Beijing Advanced Innovation Center for Big Data-Based Precision Medicine, School of Engineering Medicine, Beihang University, Beijing, China; ^5^Key Laboratory of Big Data-Based Precision Medicine (Beihang University), Ministry of Industry and Information Technology of the People's Republic of China, Beijing, China

**Keywords:** FDG-PET, anti-LGI1 encephalitis, independent component analysis, machine learning, multivariate cross-classification

## Abstract

**Objective:**

This study aims to detect the invisible metabolic abnormality in PET images of patients with anti-leucine-rich glioma-inactivated 1 (LGI1) encephalitis using a multivariate cross-classification method.

**Methods:**

Participants were divided into two groups, namely, the training cohort and the testing cohort. The training cohort included 17 healthy participants and 17 patients with anti-LGI1 encephalitis whose metabolic abnormality was able to be visibly detected in both the medial temporal lobe and the basal ganglia in their PET images [completely detectable (CD) patients]. The testing cohort included another 16 healthy participants and 16 patients with anti-LGI1 encephalitis whose metabolic abnormality was not able to be visibly detected in the medial temporal lobe and the basal ganglia in their PET images [non-completely detectable (non-CD) patients]. Independent component analysis (ICA) was used to extract features and reduce dimensions. A logistic regression model was constructed to identify the non-CD patients.

**Results:**

For the testing cohort, the accuracy of classification was 90.63% with 13 out of 16 non-CD patients identified and all healthy participants distinguished from non-CD patients. The patterns of PET signal changes resulting from metabolic abnormalities related to anti-LGI1 encephalitis were similar for CD patients and non-CD patients.

**Conclusion:**

This study demonstrated that multivariate cross-classification combined with ICA could improve, to some degree, the detection of invisible abnormal metabolism in the PET images of patients with anti-LGI1 encephalitis. More importantly, the invisible metabolic abnormality in the PET images of non-CD patients showed patterns that were similar to those seen in CD patients.

## Introduction

Leucine-rich glioma-inactivated 1 (LGI1) antibody encephalitis is one of the subtypes of autoimmune limbic encephalitis (ALE) that is characterized by a rapid progression of neurological and psychiatric deficits (1). It has been clinically demonstrated that the outcomes of ALE can be improved by early diagnosis and treatment (2–4). In the existing criteria, antibody testing is necessary and effective in the diagnosis of autoimmune encephalitis (1, 2). However, antibody testing is time-consuming and not easily accessible and therefore, a diagnosis based on antibody testing is likely to delay the treatment (2). In fact, as suggested by a position paper (2), a preliminary treatment can be initiated by an early assessment based on some traditional clinical characteristics and commonly used methods of diagnosis, such as magnetic resonance imaging (MRI), electroencephalography (EEG), or cerebrospinal fluid (CSF), before obtaining the results of antibody testing, which, in turn, will refine the initial diagnosis and treatment. However, the MRI results in some patients with anti-LGI1 encephalitis were normal (5–7) and positron emission tomography (PET) can increase the sensitivity to LGI1 encephalitis compared to MRI (6, 8, 9). Therefore, PET may be a prospective imaging tool for the early diagnosis of LGI1 encephalitis. Nevertheless, for a proportion of patients with anti-LGI1 encephalitis, the metabolic disorders were not yet perceptible (6). A recent study found the abnormal intensity of the PET signal in some areas within the medial temporal lobe and/or the basal ganglia for patients with autoimmune encephalitis (including many patients with anti-LGI1 encephalitis) who could not be identified by visual inspection (10). This finding implied that quantitative analysis may improve the identification of non-completely detectable (non-CD) patients.

Machine learning (ML), a multivariate analysis method, presents higher sensitivity than traditional univariate analysis (11, 12) and it is increasingly applied to medical imaging analysis, such as MRI, PET, and computed tomography (CT) (13–15). However, for the analysis of medical images based on ML, the number of samples is usually less than that of features, resulting in “overfitting” (16). To address this problem, feature selection or dimension reduction is conducted before training a classifier (17, 18). Independent component analysis (ICA) has been proved to be an effective method for dimension reduction (18), particularly for the multivariate analysis with a limited number of data samples (e.g., PET images).

The aim of this study was to use the combination of ICA and a method of multivariate cross-classification (MVCC) (19) to discriminate between patients with anti-LGI1 encephalitis whose metabolic disorders within the medial temporal lobe and the basal ganglia were not able to be visually detected from their PET images (referred to as non-CD patients) and healthy participants. We further aimed to explore the consistency in PET image features between the non-CD patients and the patients with anti-LGI1 encephalitis whose metabolic disorders within both the medial temporal lobe and the basal ganglia could be visually detected from their PET images [referred to as completely detectable (CD) patients].

## Materials and Methods

### Participants

This study recruited 33 patients with anti-LGI1 encephalitis (57.91 ± 11.91 years; 22 males), and part of them had been recruited in the study by Lv et al. (10). These 33 patients were in acute or subacute disease courses for in-patient care and were confirmed by the neurology physicians based on clinical symptoms and modified Rankin Scale score (all ≥3). The inclusion criteria (10) for patients are as follows: (1) the LGI1 antibodies were positive in serum and/or CSF; (2) patients presented clinical symptoms of the medial temporal lobe damage (such as drug-resistant epilepsy, cognitive impairment, and behavioral abnormalities), sleep, and autonomic dysfunctions (20); (3) PET/CT images were available; and (4) new-onset seizures showing response to immunomodulatory therapies. The exclusion criteria (10) for cases are as follows: (1) patients with acute infectious encephalitis; (2) patients with seizures caused by severe metabolic abnormalities, such as renal or hepatic failure, malignant hypertension, or severe hypo/hyperglycemia; (3) patients with seizures caused by brain structural lesions, such as stroke and tumor. We also recruited 33 healthy participants (54.64 ± 7.44 years; 23 men) without any neurologic diseases or psychiatric illnesses, who had been recruited in the study by Lv et al. (10). Table 1 summarizes the demographics of the participants. There was no significant difference in age or gender between patients with anti-LGI1 encephalitis and healthy participants (*p* > 0.05). All participants underwent ^18^Fluorodeoxyglucose (^18^F-FDG) PET/CT scan in our tertiary epilepsy center (May 2014 to November 2018). The Medical Ethics Committee of Beijing Tiantan Hospital of Capital Medical University approved this study in accordance with the Declaration of Helsinki. All participants provided written informed consent before participating in the study.

**Table 1:** Demographics of participants.

	**Age (years)**		**Gender**	
	**Range**	**Mean ±*SD***	**Male**	**Female**
Patients with anti-LGI1 encephalitis (*n* = 33)	31–78	57.91 ± 11.91	22	11
Healthy participants (*n* = 33)	40–69	54.64 ± 7.44	23	10
*p* value	0.19^a^		1.00^b^	

*LGI1, leucine-rich glioma-inactivated 1; SD, standard deviation*.

a *Two-sample Student's t-test*.

b *Fisher's exact test*.

### Image Acquisition

For each participant, a brain ^18^F-FDG PET/CT scan was performed in order to evaluate the metabolism. Blood glucose level was confirmed to be normal after a fast of at least 6 h. Then, 0.10–0.15 mCi of ^18^F-FDG per kg of body weight was injected. PET/CT images were acquired using a multidetector helical PET/CT scanner (Discovery 690, GE Medical Systems) after 30 min of rest in a dark room with eyes opened. To avoid the effect of seizures, video EEG was used to monitor brain activity to ensure the absence of seizures 1–2 h before the PET/CT scanning. In addition, before and after the PET/CT scanning, the physician observed the participant's status and confirmed the absence of seizures. Thus, during the PET/CT image acquisition, none of the patients presented seizures.

### Visual Assessment

Anti-LGI1 encephalitis is reported to be related to abnormal metabolism of the medial temporal lobe (left and/or right) (2, 21, 22). Additionally, many previous studies found that the metabolism of the basal ganglia was also abnormal for patients with anti-LGI1 encephalitis (21–24). Therefore, in the PET image of the patient with anti-LGI1 encephalitis, the medial temporal lobe and the basal ganglia of the brain were reviewed blindly and independently by an attending doctor of nuclear medicine and two experienced neurology specialists to inspect whether or not the glucose metabolism of these brain areas was abnormal. Inconsistent diagnoses were reconciled by discussion among the reviewers. Regardless of hemispheres, the patients with visible metabolic abnormality in both the medial temporal lobe and the basal ganglia in PET images were referred to as “CD patients” and those without were referred to as “non-CD patients.”

### Image Preprocessing

All image preprocessing was performed using Statistical Parametric Mapping software (SPM12, Wellcome Trust Center for Neuroimaging, London, United Kingdom; http://www.fil.ion.ucl.ac.uk/spm/software/spm12/). First, the CT images were co-registered to the corresponding PET images, and then the co-registered CT images were normalized into the Montreal Neurological Institute (MNI) template. The CT normalization was performed using an open-source Clinical Toolbox (https://www.nitrc.org/projects/clinicaltbx/), which is used as a plug-in in SPM12 (25). Second, using the transformation of CT image spatial normalization, the PET images were normalized into the MNI template. The PET images were then resampled to 2 × 2 × 2 mm^3^. Third, the normalized PET images were spatially smoothed using an 8-mm isotropic full width at half maximum (FWHM). Fourth, each smoothed PET image was normalized by dividing the intensity of each voxel by the average of the intensities across the highest 20% of the voxels whose intensities were greater than one-eighth of the mean of the PET image (26, 27). Finally, the gray matter voxels in PET images were reserved using a gray matter binary mask that was produced based on a mask of gray matter included in SPM12.

### Independent Component Analysis

**Figure 1:**
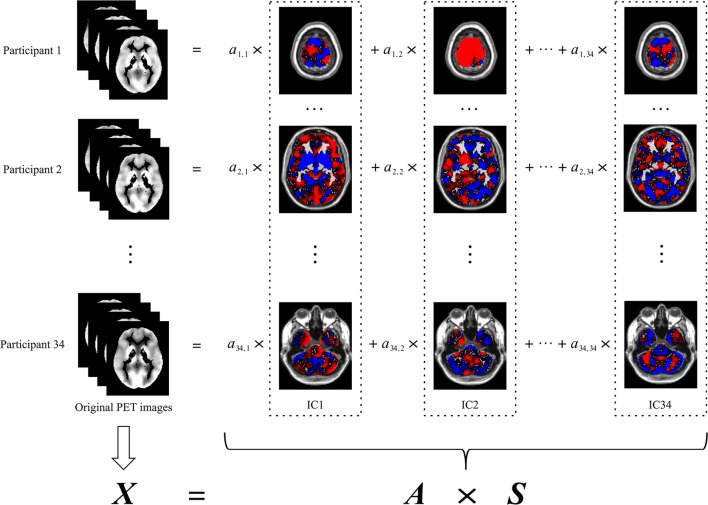
Spatial ICA decomposition of original PET images. The left column indicates the original PET image for each participant. The images in each dash rectangles indicate the corresponding independent component, which is invariant to all participants. The coefficients of each row are specific to the participant of the same row, which is used in the subsequent construction of the classification model in place of the original PET image of the corresponding participant. The coefficients of all participants, namely, *a*_*i, j*_ (*i* = 1,2,…,34; *j* = 1,2,…,34), constitute the coefficient matrix (i.e., ***A***). ***X*** denotes the original PET images, each row of which corresponds to an original PET image reshaped into a vector. ***S*** denotes the independent components, each row of which corresponds to an independent component reshaped into a vector. ICA, independent component analysis; IC, independent component.

In this study, the preprocessed PET image of each participant was the original PET image. Spatial ICA decomposed the original PET images of all participants into spatially independent components with the same resolution and size as the original PET images. As shown in Figure 1, each original PET image was the summation of the products of the independent components and their respective coefficients. The independent components were invariant to all participants, whereas the coefficients were specific to each of the participants. Thus, the original PET image could be represented by the corresponding coefficients. Because the number of these coefficients was less than the dimension of the original PET image, these coefficients were used in the subsequent construction of the classification model in place of the original PET image. The coefficients corresponding to all participants constituted the coefficient matrix (i.e., the matrix ***A*** in Figure 1).

**Figure 2:**
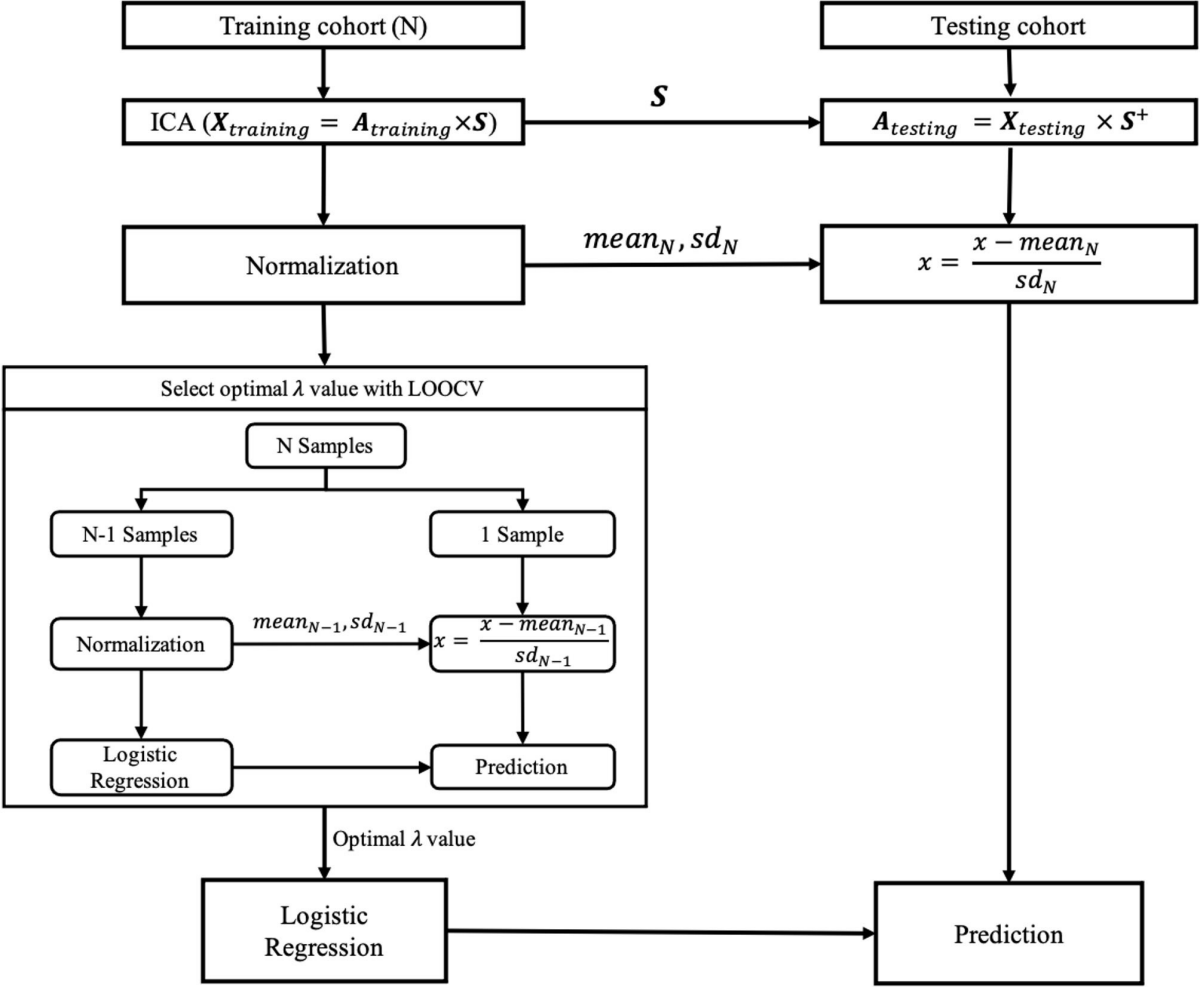
The training and testing of the classification model. ***X***_training_ and ***X***_testing_ denote the original PET images of training and testing cohorts, respectively, each row of which corresponds to an original PET image reshaped into a vector. ***A***_training_ and ***A***_testing_ are the coefficient matrixes for training and testing cohorts, respectively. ***S*** denotes the independent components in the training cohort, each row of which corresponds to an independent component reshaped into a vector. ***S***^+^, the pseudo inverse of the ***S***; SD, standard deviation; LOOCV, leave-one-out cross-validation; λ, the hyper-parameter of the logistic regression model.

In this study, spatial ICA was performed using the ICASSO toolbox included in GIFT (http://trendscenter.org/software/). Specifically, in the training cohort, the original PET images were decomposed into the coefficient matrix (**A**_training_) and 34 spatially independent components (***S***) by ICA (Figure 2). To ensure the reliability of the spatially independent components, the decomposition based on ICA was repeated 200 times. Then, all independent components were clustered according to their mutual similarities. For each cluster, the independent component that showed the maximum similarity to the other independent components in the same cluster was considered a more reliable independent component. Thus, 34 reliable independent components from 34 clusters were selected, respectively. Then, the coefficient matrix of the testing cohort (**A**_testing_) was calculated by the production of the original PET images of the testing cohort and the pseudo inverse of the ***S*** obtained in the training cohort (Figure 2). The rows of **A**_training_ and **A**_testing_ were used as the feature vectors to train and test the classification model, respectively.

The coefficients of each column of **A**_training_ were normalized by first removing their mean value and then dividing them by their *SD*. In contrast, the coefficients of each column of **A**_testing_ were normalized by first removing the mean value and then dividing by the *SD* across the coefficients of the corresponding column of **A**_training_.

For the normalized **A**_training_ and **A**_testing_, the coefficients of each row were specific to the corresponding original PET images in the training and testing cohorts and, therefore, were used in the subsequent training and testing of classification models in place of those corresponding original PET images, respectively.

### Multivariate Cross-Classification

In this study, the logistic regression model was used to discriminate between non-CD patients and healthy participants. In total, 33 healthy participants were randomly divided into two groups. There was no significant difference in age between the two groups [*t*_(31)_ = 1.59; *p* > 0.05]. The training cohort included all CD patients (*n* = 17; 57.53 ± 11.76 years; 12 men) and one group of healthy participants (*n* = 17; 56.59 ± 9.80 years; 13 men) as controls. The testing cohort included all non-CD patients (*n* = 16; 58.31 ± 12.45 years; 10 men) and another group of healthy participants (*n* = 16; 52.56 ± 2.61 years; 10 men) as controls. There was no significant difference in age between patients and healthy participants for the training cohort [*t*_(32)_ = 0.25; *p* > 0.05] or for the testing cohort [*t*_(30)_ = 1.81; *p* > 0.05]. Table 2 lists the clinical characteristics of the CD patients in the training cohort and those of the non-CD patients in the testing cohort. As summarized in Table 2, when comparing CD patients of the training cohort with the non-CD patients of the testing cohort, a significant difference was observed only in the number of MRI abnormalities of the medial temporal lobe (*p* < 0.05). In contrast, there was no significant difference in each of the other clinical characteristics between the CD patients of the training cohort and the non-CD patients of the testing cohort (*p* > 0.05). Such differences in clinical characteristics were assessed using the two-sample Student's *t*-test for continuous data and Fisher's exact test for categorical data. The two-sample Student's *t*-test was performed using the SPSS Statistics software (SPSS for macOS, version 26.0, Chicago, IL, United States), and Fisher's exact test was performed using Python, version 3.6.

**Table 2:** Comparison of clinical characteristics between CD patients of the training cohort and non-CD patients of the testing cohort.

	**CD patients**	**Non-CD patients**	***p* value**
Age (years)	57.53 ± 11.76	58.31 ± 12.45	0.85^a^
Gender (male)	12 (70.59%)	10 (62.50%)	0.72^b^
Interval time (weeks)	16.00 ± 15.45	17.56 ± 16.50	0.78^a^
Treatment, *n* (%)	7 (41.18%)	6 (37.50%)	1.00^b^
MRI abnormalities, *n* (%)			
Total	12 (70.59%)	6 (37.50%)	0.08^b^
Only MTL	12 (70.59%)	5 (31.25%)	0.04^b^
Only BG	0	1 (6.25%)	0.48^b^
Both MTL and BG	0	0	1.00^b^
Clinical symptoms, *n* (%)			
Seizures	17 (100%)	16 (100%)	1.00^b^
FBDS	7 (41.18%)	7 (43.75%)	1.00^b^
Temporal lobe seizures	9 (52.94%)	7 (43.75%)	0.73^b^
Other types	5 (29.41%)	6 (37.50%)	0.72^b^
Memory loss	3 (17.65%)	5 (31.25%)	0.44^b^
Sleep disorder	0	1 (6.25%)	0.48^b^
Headache	1 (5.88%)	0	1.00^b^
Psychiatric symptoms	1 (5.88%)	3 (18.75%)	0.34^b^
Hallucinations	0	1 (6.25%)	0.48^b^

*MTL, the medial temporal lobe; BG, the basal ganglia; FBDS, faciobrachial dystonic seizures; CD, completely detectable; non-CD, non-completely detectable*.

a *Two-sample Student's t-test*.

b *Fisher's exact test*.

As mentioned earlier, each row of the **A**_training_ and the **A**_testing_ was specific to the corresponding original PET image in the training and testing cohorts, respectively. Thus, the rows of **A**_training_ and **A**_testing_ were used as the feature vectors to train and test the logistic regression model, respectively. To determine the optimal value of the hyperparameter (λ) of the logistic regression model, leave-one-out cross-validation (LOOCV) was performed for each of 21 potential values [i.e., λ = (2^−10^, 2^−9^,..., 2^9^, 2^10^)]. The optimal λ value with the highest area under the curve (AUC) value was selected, with which a final logistic regression model was constructed using all samples from the training cohort, and then was tested by the testing cohort. The logistic regression model was implemented using scikit-learn version 0.23.2 (https://scikit-learn.org/stable/index.html).

Given the labels one and zero for patients and healthy participants in the classification model, respectively, 0.5 was set as a classification threshold. Thus, a sample with the prediction probability > 0.5 was classified as a patient, and that with the prediction probability ≤ 0.5 was classified as a healthy participant. In the testing cohort, the accuracy was calculated by dividing the number of correct predictions of the testing cohort by the number of all samples of the testing cohort. The sensitivity was calculated by dividing the number of correct predictions of the non-CD patients by the number of all non-CD patients. The specificity was calculated by dividing the number of correct predictions of controls by the total number of controls. A receiver operating characteristic (ROC) curve, which is independent of the classification threshold, was also used to evaluate the performance of the classification model. The ROC curve was plotted with the true-positive rate (i.e., sensitivity) and false-positive rate (i.e., 1-specificity) as vertical and horizontal coordinates, respectively, both of which varied as the functions of the classification threshold. The AUC was defined by the area under the ROC curve.

### Significant Independent Components Selection

In the logistic regression model, the weights corresponded to the independent components one by one, respectively. The absolute value of each weight indicated the contribution of the corresponding independent component to discriminate between patients and healthy participants. Thus, we first ranked all weights according to their absolute values and then selected the top two weights (i.e., about the top 5% or *p* < 0.05). Two independent components corresponding to these top two weights were selected as significant independent components, which presented the difference in PET images between patients and healthy participants. The significant regions of these two independent components can be identified by converting them to z-score maps.

However, in this study, the interpretation of the z-score maps should be done by considering the signs of the weights in the classification model to which these z-score maps corresponded, respectively. This is because the sign of the weight in the classification model indicated the association between the z-score map and the signal of the PET image. For example, if the weight was positive, then the positively significant regions in the z-score map indicated increased PET signals and therefore hypermetabolism, and the negatively significant regions indicated hypometabolism for patients. However, if the weight was negative, then the positively significant regions in the z-score map indicated hypometabolism, and the negatively significant regions indicated hypermetabolism for patients.

Thus, for ease of understanding, two significant independent components were first multiplied by the signs of their respective weights in the classification model. Then, these two sign-corrected independent components were converted to z-score maps, and the clusters with |z| > 2.58 (*p* < 0.01) and cluster extent ≥50 voxels were identified as significant clusters. Thus, for the patients with anti-LGI1 encephalitis, the positively significant and negatively significant regions in the z-score map indicated hypermetabolism and hypometabolism, respectively.

## Results

### Visual Assessment Results

For all 17 CD patients, abnormal metabolism was found in both the medial temporal lobe and the basal ganglia in their PET images. Table 3 summarizes the results of visual assessment for non-CD patients. Among the 16 non-CD patients, the abnormal metabolism was not able to be identified in the medial temporal lobe or the basal ganglia for seven patients and was able to be identified only in the medial temporal lobe for four patients and only in the basal ganglia for five patients.

**Table 3:** The patients with anti-LGI1 encephalitis who were non-completely detectable by visual assessment (non-CD patients).

**Patient no**.	**Visual assessment**	
	**Medial temporal lobe**	**Basal ganglia**
Patient 1	Yes	No
Patient 2	No	Yes
Patient 3	No	No
Patient 4	No	Yes
Patient 5	No	Yes
Patient 6	Yes	No
Patient 7	Yes	No
Patient 8	No	No
Patient 9	No	No
Patient 10	No	No
Patient 11	Yes	No
Patient 12	No	No
Patient 13	No	No
Patient 14	No	Yes
Patient 15	No	No
Patient 16	No	Yes

*Yes: the abnormal metabolism was able to be detected by visual assessment*.

*No: the abnormal metabolism couldn't be identified by visual assessment*.

### Multivariate Cross-Classification

**Figure 3:**
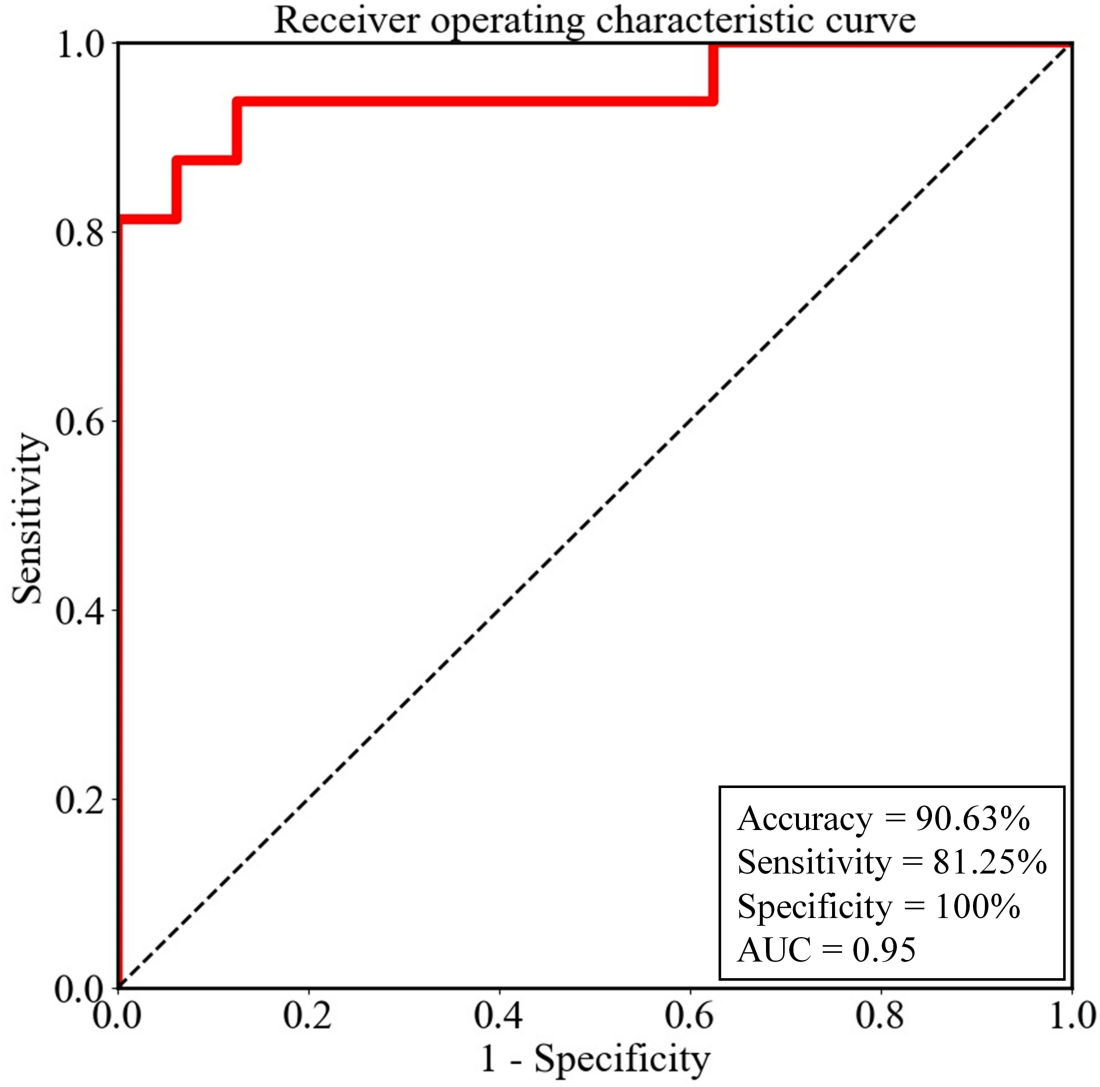
The performance of the logistic regression model for the testing cohort was evaluated by the receiver operating characteristic (ROC) curve. AUC, the area under the ROC curve.

In the training cohort, 2^−10^ was selected as the optimal λ value by using LOOCV. The accuracy was 100% in the training cohort. In the testing cohort, the constructed classifier had good generalization ability, with a sensitivity of 81.25%, a specificity of 100%, an overall accuracy of 90.63%, and an AUC value of 0.95. Thus, four non-CD patients, whose abnormal metabolism was able to be visually identified only in the medial temporal lobe, and five non-CD patients, whose abnormal metabolism was able to be visually identified only in the basal ganglia, were fully detected by our model. In contrast, only four out of seven non-CD patients, whose abnormal metabolism was not able to be visually identified in the medial temporal lobe or the basal ganglia, were detected by our model. The ROC curve of the testing cohort is shown in Figure 3.

### Significant Independent Components for Classifier

**Figure 4:**
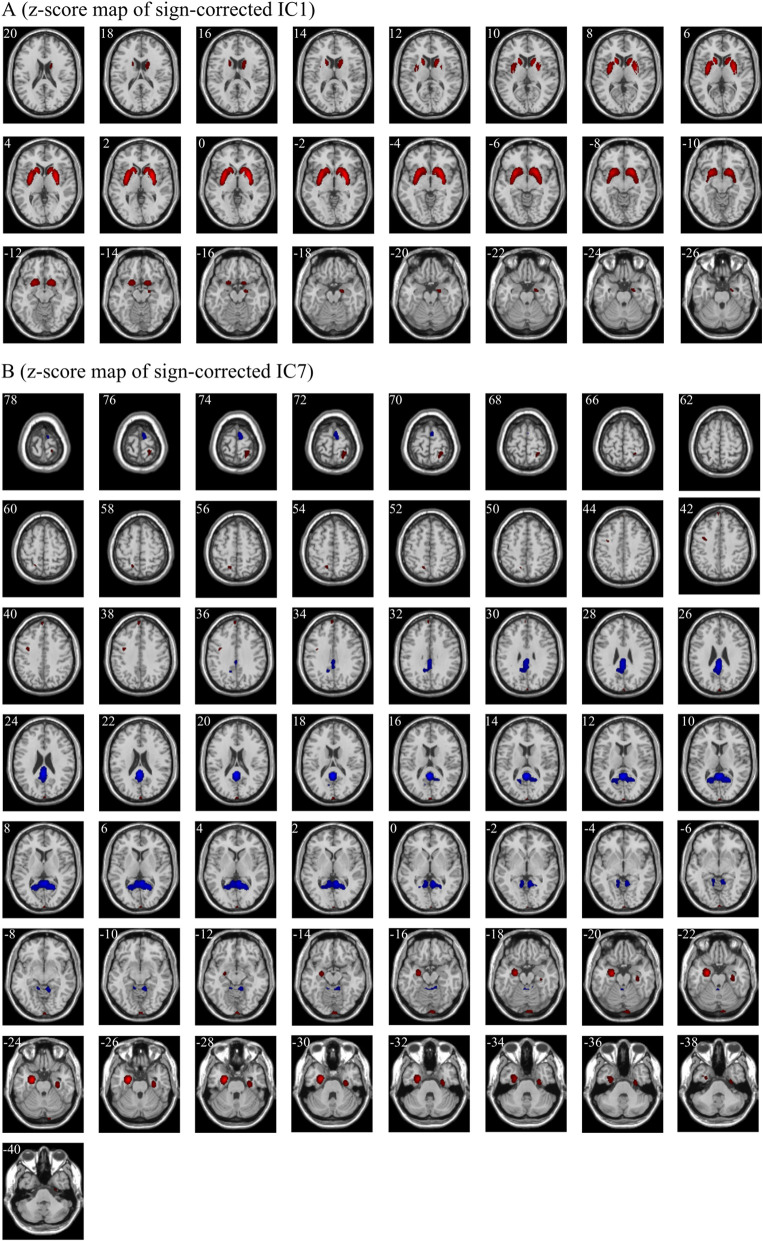
The significant regions in the z-score maps of sign-corrected IC1 **(A)** and sign-corrected IC7 **(B)** (|z| > 2.58, *p* < 0.01, cluster extent ≥ 50 voxels). Red indicates the regions with a *z* value > 2.58 and a cluster extent ≥50 voxels, and blue indicates the regions with a *z* value < −2.58 and a cluster extent ≥50 voxels. The positively significant regions (red) and the negatively significant regions (blue) indicate hypermetabolism and hypometabolism in patients with anti-LGI1 encephalitis, respectively. The white number in the upper left of each sub-figure indicates the Montreal Neurological Institute coordinates of transversal slices. R/L, right/left.

There were a total of 34 weights in the logistic regression model, which corresponded to the independent components one by one, respectively (Supplementary Table 1). We ranked all weights according to their absolute values. As shown in Supplementary Figure 1, the absolute values of the first weight and the seventh weight were evidently larger than those of the other weights. Thus, IC1 and IC7 were selected as significant independent components. Then, IC1 and IC7 were multiplied by the signs of their respective weights in the classification model. The z-score maps of the sign-corrected IC1 and the sign-corrected IC7 were shown in Figure 4. For each of these two z-score maps, all local peaks within each significant cluster and their respective corresponding brain regions are listed in Table 4. As shown in Figure 4 and summarized in Table 4, significantly increased PET signals were observed in the bilateral medial temporal lobe, the bilateral basal ganglia, the left precuneus, the left medial part of the superior frontal gyrus, the right postcentral gyrus, and the left calcarine fissure and surrounding cortex, indicating that the patients with anti-LGI1 encephalitis presented hypermetabolism in these brain regions. In contrast, significantly decreased PET signals were observed in the right supplementary motor area, the bilateral calcarine fissure and surrounding cortex, and the lobule III of the vermis, indicating that the patients with anti-LGI1 encephalitis presented hypometabolism in these brain regions. These findings suggest that abnormal metabolism of these brain regions played an important role in discriminating between the patients with anti-LGI1 encephalitis and healthy participants.

**Table 4:** The significant brain regions (|z| > 2.58, *p* < 0.01, cluster extent ≥ 50 voxels) included in the two sign-corrected independent components corresponding to the first two weights in the rank of the absolute values of all weights of the logistic regression model.

**IC**	**Cluster**	**Peak level**				**Brain regions**	
		***z* value**	**x (mm)**	**y (mm)**	**z (mm)**	**L/R**	**Name**
IC1	101	3.73	20	−10	−18	R	Medial temporal lobe (Hippocampus)
(−17.90^*^)	1,796	6.98	−16	16	4	L	Basal ganglia (Caudate nucleus)
		7.74	−32	−6	0	L	Basal ganglia (Putamen)
		7.94	−30	0	0	L	Basal ganglia (Putamen)
		8.18	−26	8	−4	L	Basal ganglia (Putamen)
	1,860	9.35	26	6	0	R	Basal ganglia (Putamen)
IC7	51	3.21	−14	−58	54	L	Precuneus
(14.93^*^)	59	3.41	−2	58	38	L	Superior frontal gyrus (medial part)
	62	3.65	−34	−2	38	–	–
	146	−4.15	8	−2	74	R	Supplementary motor area
	151	3.89	26	−44	72	R	Postcentral gyrus
	244	3.57	2	−102	10	L	Calcarine fissure and surrounding cortex
		3.64	2	−100	18	–	–
		4.03	4	−102	−8	–	–
		4.33	8	−98	−18	–	–
		4.44	4	−100	−14	–	-
	308	5.37	32	−18	−26	R	Medial temporal lobe (Parahippocampal gyrus)
	750	10.07	−24	−6	−26	L	Medial temporal lobe (Hippocampus)
	2,816	−6.34	0	−48	18	–	–
		−5.01	20	−56	8	R	Calcarine fissure and surrounding cortex
		−4.25	−18	−58	8	L	Calcarine fissure and surrounding cortex
		−2.74	−2	−46	−20	–	Lobule III of vermis

*IC, independent component; -, there were no relevant results; R/L, right/left*.

* *The weight corresponding to the independent component*.

Additionally, there was also an obvious cutoff of the absolute value of weight between the top seven weights and the other ones. The z-score maps of the sign-corrected independent components corresponding to these weights (except the top two weights) are shown in Supplementary Figure 2, and the significant regions of these sign-corrected independent components are summarized in Supplementary Table 2.

## Discussion

In this study, an MVCC method combined with ICA was used to analyze the PET data to detect non-CD patients and explore the consistency in PET image features between non-CD patients and CD patients. To the best of our knowledge, this is the first study in which the MVCC method combined with ICA was used to analyze PET images of patients with anti-LGI1 encephalitis. In the MVCC, a logistic regression model was first trained using the PET images of CD patients and then tested using the PET images of non-CD patients. By transferring the learning from the former to the latter, the MVCC can detect the relatively weak PET signal changes related to anti-LGI1 encephalitis of non-CD patients and therefore discriminate between non-CD patients and healthy participants with higher accuracy. Compared to visual assessment, the MVCC increased the sensitivity of the detection of non-CD patients and, at the same time, preserved the highest specificity. However, it should be noted that for the non-CD patients whose abnormal metabolism was not able to be visually identified in the medial temporal lobe or the basal ganglia, only four out of seven (about 57.14%) were detected by our ML method. One probable reason for this relatively low detection rate of this type of patient may be that there was no such type of patient in the training cohort. Thus, in future studies, more patients without visible metabolic abnormalities in the medial temporal lobe or the basal ganglia should be included in the training cohort to improve the performance of the classification model.

In this study, the important roles of ICA were feature extraction and dimension reduction. The PET image of the patient with anti-LGI1 encephalitis is actually the compound of multiple different patterns of PET signals resulting from different sources, such as metabolic abnormality related to anti-LGI1 encephalitis, other brain activities, noise, and background. Among these patterns of PET signals, if the one related to anti-LGI1 encephalitis metabolic abnormality is relatively strong, it will be visible in PET images (i.e., CD patient). However, if this pattern of PET signal is relatively weak, it will be covered by the superposition of the other patterns of PET signals and therefore is not able to be detected by visual assessment (i.e., non-CD patient). In this study, these overlapping patterns of PET signals related to different sources were separated by ICA into different spatially independent components. As revealed by the results of MVCC, the independent components with PET signal changes in the medial temporal lobe and the basal ganglia provided the most contribution to the discrimination between non-CD patients and healthy participants. Thus, the important findings of the present study were that non-CD patients presented similar patterns of metabolic abnormality as those of CD patients, though the PET signals related to anti-LGI1 encephalitis metabolic abnormality were unable to be detected in them. These findings are consistent with previous studies about patients with anti-LGI1 encephalitis (6, 28, 29), suggesting that anti-LGI1 encephalitis is closely related to the metabolic abnormality in the medial temporal lobe and the basal ganglia. A recent study (7) also found that patients with anti-LGI1 encephalitis presented hypermetabolism in the medial temporal lobe and the basal ganglia (i.e., including the putamen and the caudate), consistent with the findings of the present study. However, some other brain regions showing metabolic abnormalities in the study (7) (e.g., angular gyrus, olfactory, and pons) were observed as normal regions in the present study. It should be noted that, in the study (7), the mean of standardized uptake values across the regions of interest was used to measure the metabolic abnormalities. In contrast, in this study, the independent images related to anti-LGI1 encephalitis were separated by ICA. This difference in the methods of feature extraction may be one of the potential reasons for the discrepancies in the findings between these studies.

In addition to the medial temporal lobe and the basal ganglia, other brain regions of patients with anti-LGI1 encephalitis were also revealed to present different PET signals from those of the healthy participants (Table 4). A previous study found that patients with anti-LGI1 encephalitis presented abnormal metabolism in the precuneus (23). A recent study reported that the supplementary motor area of two patients with anti-LGI1 encephalitis presented hypometabolism in their PET images (30). This existing evidence along with our findings suggests that the metabolic abnormality of the left precuneus and the right supplementary motor area may also play important roles in anti-LGI1 encephalitis.

As for the left medial part of the superior frontal gyrus, the right postcentral gyrus, the bilateral calcarine fissure and surrounding cortex, and the lobule III of the vermis, they are rarely specifically reported by previous studies about anti-LGI1 encephalitis. These brain regions are a part of the frontal lobe, the parietal lobe, the occipital lobe, and the cerebellum, respectively. The existing studies using PET have reported that metabolic abnormalities were observed in these lobes of the patients with anti-LGI1 encephalitis, for example, the bilateral frontal lobe (8), the bilateral (8) and right (31) parietal lobe, the occipital lobe (8, 29) and the cerebellum (29). Thus, these brain regions with abnormal PET signals in the present study may also be related to the anti-LGI1 encephalitis. In the present study, the mixed PET signals were separated by ICA and then analyzed as a whole by the classifier. Additionally, as confirmed in the present study, the multivariate analysis is more sensitive to the changes in PET signals than visual assessment. Thus, compared to conventional visual assessment, the MVCC method combined with ICA can reveal more potential brain regions with metabolic abnormalities related to anti-LGI1 encephalitis. However, the roles of these brain regions in anti-LGI1 encephalitis need to be further explored.

The present study still had some limitations. First, the number of samples was small. This is because the prevalence and incidence of anti-LGI1 encephalitis are relatively low (32, 33). Further work will include as many as possible patients with anti-LGI1 encephalitis, particularly the data from multiple institutes to improve the performance of the classifier. Second, the good performance may be partly due to the involvement of the patients with visible abnormal metabolism either only in the medial temporal lobe or only in the basal ganglia in the testing cohort. Thus, more patients whose abnormal metabolism was not able to be visually identified in either the medial temporal lobe or the basal ganglia should be included in future studies to improve the ability of the classification model to identify this type of patient.

In general, as a relatively new imaging methodology, ^18^F-FDG-PET has presented a wide application prospect in the diagnosis of autoimmune encephalitis (34). This study used an MVCC method of PET imaging based on ICA and logistic regression, which was able to take the best advantage of the information of PET images to reveal the difference in PET signals between the patients with anti-LGI1 encephalitis and healthy participants, even in cases where this difference is not accessible with visual assessment. Our method is helpful to promote the application of PET imaging in the early diagnosis of autoimmune encephalitis, whose clinical effectiveness needs to be further validated by many prospective studies.

## Conclusion

This study used an MVCC method combined with ICA to detect non-CD patients and explore the consistency in PET image features between non-CD patients and CD patients. This method can improve, to some degree, the detection of invisible abnormal metabolism in the PET images of patients with anti-LGI1 encephalitis. More importantly, this study suggested that the patterns of PET signal changes caused by metabolic abnormalities associated with anti-LGI1 encephalitis were similar in CD patients and non-CD patients.

## Data Availability Statement

The original contributions presented in the study are included in the article/Supplementary Material, further inquiries can be directed to the corresponding authors.

## Ethics Statement

The studies involving human participants were reviewed and approved by the Medical Ethics Committee of Beijing Tiantan Hospital of Capital Medical University. The patients/participants provided their written informed consent to participate in this study.

## Author Contributions

JP and JL contributed to the conception and design of the study and drafted the manuscript. JP, GZ, and RS contributed to the analysis of data. RL, QW, and XZ contributed to the acquisition of data. JL, RL, XZ, and LA revised the manuscript. JL and LA supervised the study. All authors contributed to the study conception and design. All authors contributed to the article and approved the submitted version.

## Funding

This study was supported by grants from the Beijing Natural Science Foundation (Grant Nos. Z200027 and 7192054), the Application Research of Capital Clinical Characteristics (Grant No. Z181100001718082), the Beijing Dongcheng District Outstanding Talent Funding Project (Grant No. 2019DCT-M-18), the National Natural Science Foundation of China (Grant No. 81771143), and the National Key Research and Development Program of China (Grant No. 2018YFC1315201).

## Conflict of Interest

The authors declare that the research was conducted in the absence of any commercial or financial relationships that could be construed as a potential conflict of interest.

## Publisher's Note

All claims expressed in this article are solely those of the authors and do not necessarily represent those of their affiliated organizations, or those of the publisher, the editors and the reviewers. Any product that may be evaluated in this article, or claim that may be made by its manufacturer, is not guaranteed or endorsed by the publisher.
