# Supplementary material for: The Detection of Invisible Abnormal Metabolism in the FDG-PET Images of Patients With Anti-LGI1 Encephalitis by Machine Learning
Source: Front Neurol. 2022 May 30;13:812439. doi: 10.3389/fneur.2022.812439 (PMC9197115; doi:10.3389/fneur.2022.812439)
Supplement: Supplementary file 1 [file Table_1.DOCX]

***Supplementary Material***


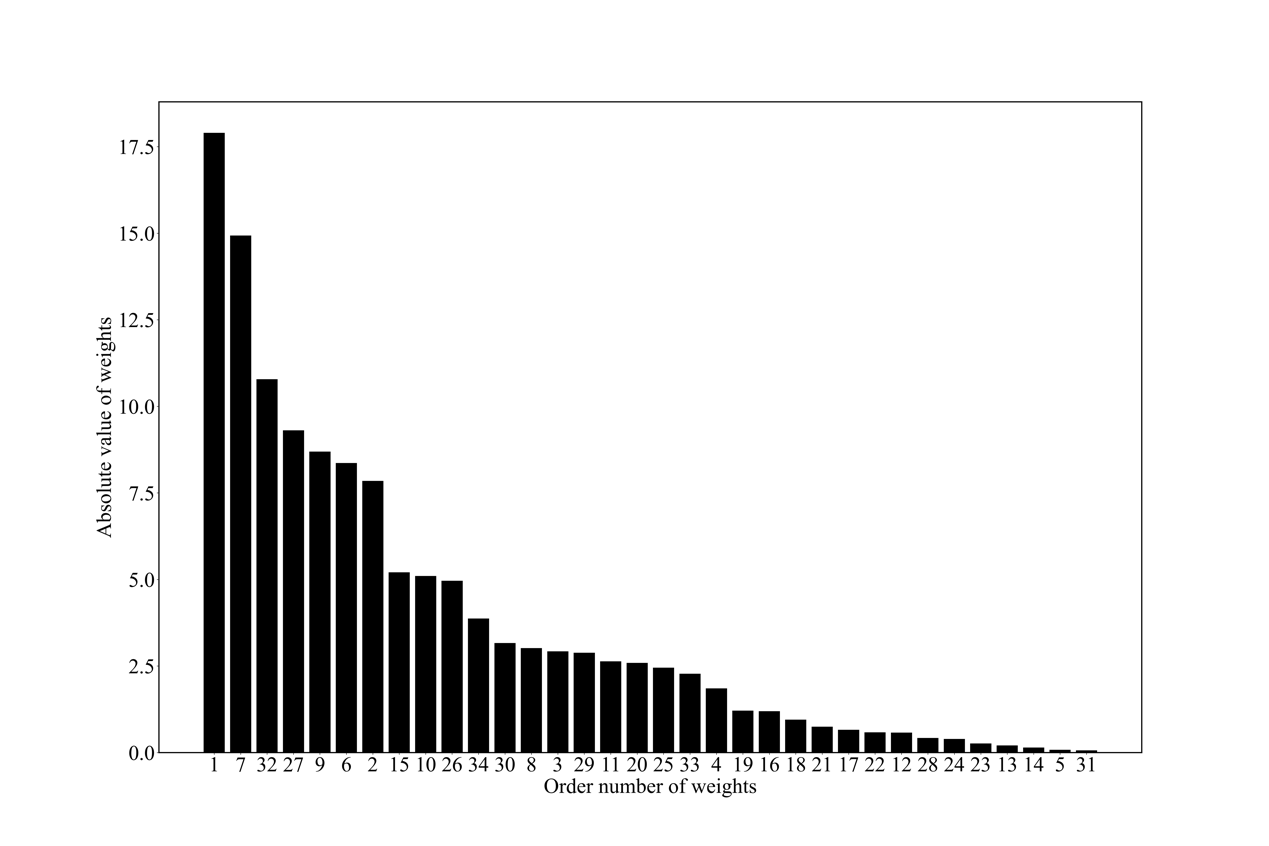


**Supplementary Figure 1.** The illustration of weights in the classification model respectively corresponding to all independent weights in a descending order. The horizontal axis indicates the order number of weights. The vertical axis indicates the absolute value of weights.


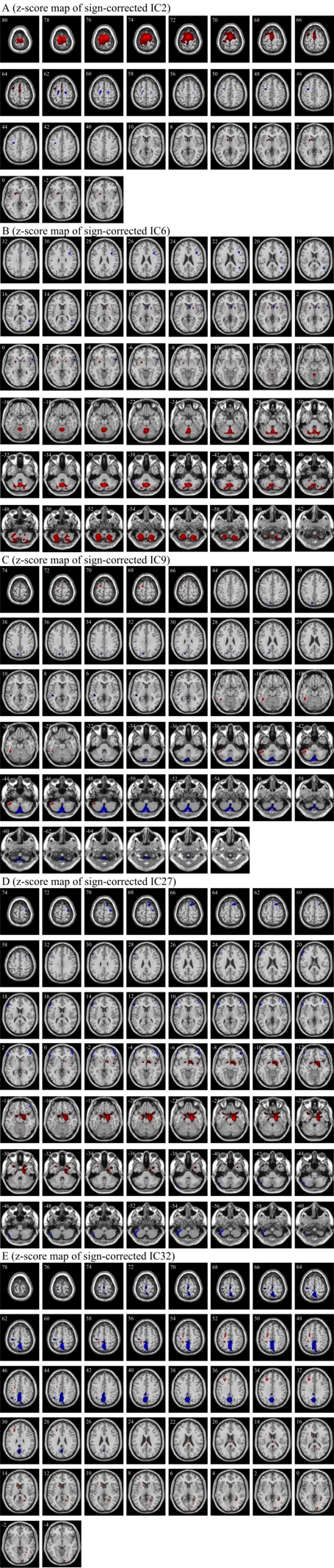


**Supplementary Figure 2.** The significant regions in the z-score maps of sign-corrected IC2 (A), sign-corrected IC6 (B), sign-corrected IC9 (C), sign-corrected IC27 (D), and sign-corrected IC32 (E) (|z| >2.58, p < 0.01, cluster extent ≥ 50 voxels). Red indicates the regions with z value > 2.58 and cluster extent ≥ 50 voxels, and blue indicates the regions with z value < -2.58 and cluster extent ≥ 50 voxels. The positively significant regions (red) and the negatively significant regions (blue) indicate hypermetabolism and hypometabolism of patients with anti-LGI1 encephalitis, respectively. The white number in the up-left of each sub-figure indicates the Montreal Neurological Institute coordinates of transversal slices. R/L: right/left.

**Supplementary Table 1.** Model weights for all 34 independent components

| Independent component | Corresponding model weight |
| --- | --- |
| IC1 | -17.90 |
| IC2 | 7.85 |
| IC3 | 2.92 |
| IC4 | 1.85 |
| IC5 | 0.08 |
| IC6 | -8.36 |
| IC7 | 14.93 |
| IC8 | -3.02 |
| IC9 | 8.69 |
| IC10 | 5.10 |
| IC11 | -2.63 |
| IC12 | 0.58 |
| IC13 | 0.21 |
| IC14 | 0.15 |
| IC15 | 5.21 |
| IC16 | 1.20 |
| IC17 | -0.66 |
| IC18 | -0.95 |
| IC19 | -1.21 |
| IC20 | -2.59 |
| IC21 | -0.75 |
| IC22 | -0.58 |
| IC23 | 0.27 |
| IC24 | 0.40 |
| IC25 | -2.45 |
| IC26 | 4.96 |
| IC27 | 9.31 |
| IC28 | -0.42 |
| IC29 | -2.88 |
| IC30 | -3.16 |
| IC31 | 0.07 |
| IC32 | 10.78 |
| IC33 | -2.28 |
| IC34 | -3.87 |

IC: independent component

**Supplementary Table 2.** The significant brain regions (|z| >2.58, p < 0.01, cluster extent ≥ 50 voxels) included in sign-corrected IC2, sign-corrected IC6, sign-corrected IC9, sign-corrected IC27 and sign-corrected IC32.

| IC | Cluster | Peak level | | | |  | Brain regions | |
| --- | --- | --- | --- | --- | --- | --- | --- | --- |
|  |  | z value | x(mm) | y(mm) | z(mm) |  | L/R | Name |
| IC2 | 66 | -4.28 | 18 | -18 | 60 |  | - | - |
|  | 82 | -3.88 | -30 | -4 | 46 |  | L | Precentral gyrus |
|  | 86 | -3.42 | -14 | -12 | 60 |  | - | - |
|  | 136 | 3.24 | -12 | 10 | 4 |  | L | Basal ganglia (Caudate nucleus) |
|  | 3231 | 9.93 | 10 | -8 | 76 |  | R | Supplementary motor area |
| IC6 | 53 | -3.35 | -54 | -48 | -42 |  | L | Crus II of cerebellar hemisphere |
|  | 64 | -3.22 | 10 | 14 | 6 |  | R | Basal ganglia (Caudate nucleus) |
|  | 76 | -3.42 | 44 | -56 | 18 |  | R | Middle temporal gyrus |
|  | 77 | 3.34 | 18 | 10 | 0 |  | - | - |
|  | 79 | -3.09 | 60 | 26 | 4 |  | R | Inferior frontal gyrus, triangular part |
|  | 93 | 3.31 | -20 | 8 | 0 |  | L | Basal ganglia (Putamen) |
|  | 117 | -3.54 | 36 | 12 | 28 |  | R | Inferior frontal gyrus, opercular part |
|  | 4837 | 5.54 | 0 | -62 | -36 |  | - | Lobule VIII of vermis |
| IC9 | 51 | 3.76 | -16 | -2 | 70 |  | L | Superior frontal gyrus, dorsolateral |
|  | 81 | -3.72 | -44 | -30 | 6 |  | L | Superior temporal gyrus |
|  | 107 | 3.55 | -44 | -46 | -18 |  | L | Inferior temporal gyrus |
|  | 120 | -3.78 | -16 | -76 | 30 |  | L | Cuneus |
|  | 170 | 3.93 | -46 | -46 | -44 |  | L | Crus II of cerebellar hemisphere |
|  | 2289 | -9.45 | -10 | -84 | -48 |  | - | - |
| IC27 | 69 | -3.56 | 30 | -14 | 72 |  | R | Precentral gyrus |
|  | 195 | -3.78 | 14 | 14 | 66 |  | R | Supplementary motor area |
|  | 304 | -3.70 | -50 | -58 | -52 |  | L | Crus II of cerebellar hemisphere |
|  | 415 | -3.76 | 46 | 54 | 4 |  | R | Middle frontal gyrus |
|  | 418 | -3.47 | -56 | 26 | 18 |  | L | Inferior frontal gyrus, triangular part |
|  | 638 | 4.00 | -18 | -16 | -26 |  | L | Medial temporal lobe (Parahippocampal gyrus) |
|  | 2875 | 10.23 | 16 | -12 | -22 |  | - | - |
| IC32 | 63 | -3.50 | -36 | -58 | 46 |  | L | Inferior parietal gyrus, excluding supramarginal and angular gyri |
|  | 67 | 3.59 | 4 | -40 | 20 |  | R | Posterior cingulate gyrus |
|  | 71 | 3.26 | -12 | 12 | 12 |  | L | Basal ganglia (Caudate nucleus) |
|  | 72 | 3.86 | -26 | -36 | 52 |  | L | Postcentral gyrus |
|  | 78 | 3.29 | 14 | -92 | 0 |  | R | Calcarine fissure and surrounding cortex |
|  | 120 | 4.44 | -24 | 28 | 32 |  | L | Superior frontal gyrus, dorsolateral |
|  | 120 | 3.91 | -22 | -4 | 50 |  | L | Superior frontal gyrus, dorsolateral |
|  | 121 | 3.28 | 28 | -54 | 2 |  | R | Lingual gyrus |
|  | 162 | -3.77 | -34 | -26 | 60 |  | L | Precentral gyrus |
|  | 3403 | -6.36 | 0 | -60 | 56 |  | - | - |

IC: independent component, R/L: right/left.
